# Supplementary material for: The integration of tuberculosis and HIV testing on GeneXpert can substantially improve access and same-day diagnosis and benefit tuberculosis programmes: A diagnostic network optimization analysis in Zambia
Source: PLOS Glob Public Health. 2023 Jan 25;3(1):e0001179. doi: 10.1371/journal.pgph.0001179 (PMC10022337; doi:10.1371/journal.pgph.0001179)
Supplement: Attachment — Submitted filename: Response to reviewers.docx [file pgph.0001179.s001.docx]

Dear Dr Lopez-Varela

Thank you for considering our paper “*The integration of tuberculosis and HIV testing on GeneXpert can substantially improve access and same-day diagnosis and benefit tuberculosis programmes: a diagnostic network optimization analysis in Zambia*” (Manuscript number: PGPH-D-22-00783). We are very grateful to the reviewers for their useful comments.

Please do consider this manuscript for the Collection - *Diagnostics in Global Health.*

In this letter we specify how we responded to each comment. Reviewer and editorial comments are italicized, with responses in plain text.

**JOURNAL REQUIREMENTS**

*1. Our staff editors have determined that your manuscript is likely within the scope of our Diagnostics in Global Health Call for Papers. This editorial initiative is headed by a team of Guest Editors for PLOS GPH: Senjuti Saha (Child Health Research Foundation, Bangladesh) and Titus Divala (Public Health Scotland, University of Glasgow and University of Malawi College of Medicine). The Collection will encompass a diverse range of research articles about diagnostics in global health, including innovation and deployment of point of care diagnostics; subsets of diagnostics related to infectious diseases, chronic diseases and injuries; policies related to and regulation of diagnostics; supply chain issues; and the affordability, accessibility, and availability of essential diagnostics.  Additional information can be found on our announcement page:* [*https://collections.plos.org/call-for-papers/diagnostics-in-global-health/*](https://collections.plos.org/call-for-papers/diagnostics-in-global-health/) *If you would like your manuscript to be considered for this collection, please let us know in your cover letter and we will ensure that your paper is treated as if you were responding to this call.  Please note that being considered for the Collection does not require additional peer review beyond the journal’s standard process and will not delay the publication of your manuscript if it is accepted by PLOS GPH. If you would prefer to remove your manuscript from collection consideration, please specify this in the cover letter.*

**Response:** Please see above. Please consider this manuscript for this collection

*2. Please amend your detailed online Financial Disclosure statement. This is published with the article. It must therefore be completed in full sentences and contain the exact wording you wish to be published.*

*a. State the initials, alongside each funding source, of each author to receive each grant.
b. Please expand the acronym “FIND” (as indicated in your financial disclosure) so that it states the name of your funders in full.*

**Response:** The Financial Disclosure statement has been amended to include the author initials and the full funder name.

*3. In the online submission form, you indicated that “Data is available on request from the Ministry of Health, Zambia. Please contact Powell Choonga (*[*powellchoonga@gmail.com*](mailto:powellchoonga@gmail.com)*).”. All PLOS journals now require all data underlying the findings described in their manuscript to be freely available to other researchers, either 1. In a public repository, 2. Within the manuscript itself, or 3. Uploaded as supplementary information.
This policy applies to all data except where public deposition would breach compliance with the protocol approved by your research ethics board. If your data cannot be made publicly available for ethical or legal reasons (e.g., public availability would compromise patient privacy), please explain your reasons by return email and your exemption request will be escalated to the editor for approval. Your exemption request will be handled independently and will not hold up the peer review process, but will need to be resolved should your manuscript be accepted for publication. One of the Editorial team will then be in touch if there are any issues.*

**Response: Still to be confirmed with country team**

*4. Please remove your figures from within your manuscript file, leaving only the individual TIFF/EPS image files.  These will be automatically included in the reviewer’s PDF.*

**Response:** Figures have been removed from the manuscript file

*5. Please ensure that you refer to Tables 1, 3, and 6 in your text as, if accepted, production will need these references to link the reader to the tables.*

**Response:** These table references have been added to the text.

*6. We do not publish any copyright or trademark symbols that usually accompany proprietary names, eg (R), (C), or TM (e.g. next to drug or reagent names). Please remove all instances of trademark/copyright symbols throughout the text, including ® (GeneXpert®) on page 23.*

**Response:** These have been removed from the manuscript text.

*7. All figures and supporting information files will be published under the Creative Commons Attribution License (creativecommons.org/licenses/by/4.0/). Authors retain ownership of the copyright for their article and are responsible for third-party content used in the article.*

*Figures 1 and 2: please (a) provide a direct link to the base layer of the map used and ensure this is also included in the figure legend; (b) provide a link to the terms of use / license information for the base layer. We cannot publish proprietary or copyrighted maps (e.g. Google Maps, Mapquest) and the terms of use for your map base layer must be compatible with our CC-BY 4.0 license.*

*If your map was obtained from a copyrighted source please amend the figure so that the base map used is from an openly available source. Alternatively, please provide explicit written permission from the copyright holder granting you the right to publish the material under our CC-BY 4.0 license.*

*Please note that the following CC BY licenses are compatible with PLOS license: CC BY 4.0, CC BY 2.0 and CC BY 3.0, meanwhile such licenses as CC BY-ND 3.0 and others are not compatible due to additional restrictions.*

*If you are unsure whether you can use a map or not, please do reach out and we will be able to help you. The following websites are good examples of where you can source open access or public domain maps: 
* U.S. Geological Survey (USGS) - All maps are in the public domain. (*[*http://www.usgs.gov*](http://www.usgs.gov)*) 
* PlaniGlobe - All maps are published under a Creative Commons license so please cite “PlaniGlobe,* [*http://www.planiglobe.com*](http://www.planiglobe.com)*, CC BY 2.0” in the image credit after the caption. (*[*http://www.planiglobe.com/?lang=enl*](http://www.planiglobe.com/?lang=enl)*) 
* Natural Earth - All maps are public domain. (*[*http://www.naturalearthdata.com/about/terms-of-use/*](http://www.naturalearthdata.com/about/terms-of-use/)*)*

*Please upload any written confirmation as an 'Other' file type. It must clarify that the copyright holder understands and agrees to the terms of the CC BY 4.0 license; general permission forms that do not specify permission to publish under the CC BY 4.0 will not be accepted. Note that uploading an email confirmation is acceptable.*

**Response:** I am not sure this applies to the two maps (Figure 1 and 2) in the manuscript as they do not use any basemaps. The only thing that is used is the shapefile for administrative boundaries which was obtained from <https://data.humdata.org/faqs/licenses> which does have a CC BY license. I emailed PLoS Global Public Health on the 24 May 2022 asking advice regarding this matter.

*8. Please review your reference list to ensure that it is complete and correct. If you have cited papers that have been retracted, please include the rationale for doing so in the manuscript text, or remove these references and replace them with relevant current references. Any changes to the reference list should be mentioned in the rebuttal letter that accompanies your revised manuscript. If you need to cite a retracted article, indicate the article’s retracted status in the References list and also include a citation and full reference for the retraction notice.*

**Response:** The reference list has been reviewed. No cited papers have been retracted.

**REVIEWER 1:**

*1. Abstract
a. Lines 27 – 52: As a suggestion. it might be better if you put titles on abstract text. (Background or introduction, objective or aim, methods, results, conclusion, keywords.)*

**Response:** Thank you for the suggestion. However, the authors were following the requirements as stipulated in the submission guidelines for PLoS Global Public Health articles: “While the Abstract is conceptually divided into three sections (Background, Methodology/Principal Findings, and Conclusions/Significance), do not apply these distinct headings to the Abstract within the article file.”
 *2. Methods
a. It is not clear what kind of study. It would be better to clarify what type of cohort study, cross-sectional, retrospective, prospective, observational, or interventional?*

**Response:** This has been clarified upfront in the Methods section by the addition of the following sentence: *“Using OptiDx software and a national cohort of retrospective, cross-sectional laboratory data, we first established the baseline diagnostic network based on 2020 (October 2019 - September 2020) testing demand, referral linkages, testing sites, platforms, and costs for the HIV and TB programmes respectively”*

*b. Criteria of inclusion and exclusion are no clear?*

**Response:** Under the sub-title ‘Study setting and scope’ we define the facilities included, the laboratories, devices and test types. As noted in (c ) below, all health facilities referring TB/HIV specimens were included. Further all laboratories and devices with HIV/TB molecular testing capability are included. The following sentence has been adjusted to clarify this: “. All laboratories (277) and diagnostic devices at these laboratories with HIV/TB molecular testing capability (a total of 363) were included along with 3 tests types: HIV VL, HIV EID, and Xpert MTB/RIF.”

*c. Sample and sampling. How was the sample size selected? It seems to us that you have collected the universe of existing data, if applicable, clarify.*

**Response:** This is correct. It is all health facilities referring TB/HIV specimens and laboratories in Zambia. This is noted in ‘Study setting and scope’: *“All public and private health facilities (a total of 2,717) across all ten provinces in Zambia were included in the analysis.”.*

*d. You can clarify which variables of interest will be studied.*

**Response:** The variables of interest are described under the ‘Outcomes’ sub-section which describes how access, the variable of interest, is measured (distance travelled, proportion tested onsite, and turnaround time). Further, the subsection ‘costs’ details the different cost elements that are of interest.

*e. Lines 135 – 136: Data collection:
i.) not clear which system you used. Was it DisaLink, DisaTB? others? If there is any copyright, please indicate it.*

**Response:** The following sentence has been added to the subsection ‘test demand’ to describe in more detail what systems were used to extract test demand data. “*VL and EID demand data was obtained from DisaLab – the laboratory information system; and TB data was obtained from the Ministry of Health Health Management Information System and triangulated with National TB Programme datasets*.”

*ii.) Please explain more in detail how you did collect data on a database, how you anonymized them and how you were able to produce tables to be entered in your dataset analysis (i.e: Excel or…)*

**Response:** The following sentence has been added to the data inputs section: **“***All extracted aggregated laboratory data was compiled in excel, formatted and then uploaded into OptiDx to be further analysed.”*
 *f. Lines 231 – 237: Analysis of statistical tests.
i.) Would it be better to clarify what kind? If you are going to do descriptive statistics in MS Excel only, or if you are going to use some advanced statistical program like SPSS, STATA, R-Project, etc.
ii.) If there is any copyright, please indicate it*

**Response:** All analyses in this manuscript (including the sensitivity analysis mentioned in Lines 231-237) were conducted in OptiDx (as described Line 119 to 128).
 *3. Results:
a. The author presents four analysis tables. It would be nice if you can also divide the results into 4 subtitles followed by respective mentioned text and table, so that it is more understandable*

## Response: The following subtitles have been added to the Results section: (1) Location of capacity and testing demand at baseline, (2) Comparison of baseline and integrated scenarios, (3) Access improvements for EID, (4) Sensitivity analysis.

**REVIEWER 2:**

*This is an interesting study report assessing the potential impact of moving the current laboratory centralized priority-HIV [infants: early infant diagnosis (EID) and viral load (VL); women: pregnant/breastfeeding HIV/VL testing] testing to an integrated on GenXpert TB testing in Zambia through a network mathematical model. The authors set in the background a context of a country that has already managed to decentralized TB GenXpert testing, and there is a global availability of capacity to add other tests in the current TB GeneXpert cartridges.*

**Response:** We thank the Reviewer for their kind comment.

*Few issues:
1. We need a quick introduction of DNO [Diagnostic Network Optimization] somewhere in the background. This is new for a typical reader of Plos Global Public Health. This should be in the introduction/background; and I think it should also briefly be covered in the abstract.
It would be good to cover as well to cover the fundamental assumptions of DNO.*

**Response:** The following sentence has been added to the introduction “*DNO is a geospatial analytics approach that uses optimization techniques to model a diagnostic network and ensure the greatest access to services, whilst maximizing the overall efficiency of the system*”. A description of the fundamental assumptions of DNO, as used by the algorithm in the OptiDx tool, is detailed in the Methods – Modelled scenarios.

*2. There is an important amount of abbreviation here. Please make a list.*

**Response:** The authors have followed the requirements as stipulated in the submission guidelines for PLoS Global Public Health articles on abbreviations and have defined all abbreviations upon first appearance in the text.

*3. The information about the parameters and constraints is good. The base scenario is analysed for an entire year; therefore, no intra-year variation is considered. In many parts of Africa, during the rainy season, roads become blocked, affecting logistics and access/availability to facilities and commodities. At least discuss this.*

**Response:** Thank you for highlighting this. The following sentence has been added to the Methods section on the referral system: “*The speed of the transportation mode in OptiDx is fixed, and as such does not take into account seasonal variation and how this might change with the rainy season compromising road access and travel times between facilities and laboratories*.”

*4. About the costing, please add information as to what perspective (society or the payer) these costs are considered here.*

**Response:** Thank you for noting this. The costing perspective is the provider. This has been added to the manuscript. “*All costs are calculated from the provider-perspective*.”

*5. Abstract:
- Personally, I like to have this divided into introduction, methods, results and conclusion. So I would suggest the authors do so.*

**Response:** Thank you for the suggestion. However, the authors were following the requirements as stipulated in the submission guidelines for PLoS Global Public Health articles: “While the Abstract is conceptually divided into three sections (Background, Methodology/Principal Findings, and Conclusions/Significance), do not apply these distinct headings to the Abstract within the article file.”

*- Please add a brief statement about the DNO*

**Response:** The abstract has a word limit of 300 and unfortunately the limit has been reached. On review of the abstract, the authors were unable to reduce it further in order to accommodate a succinct description of DNO but included the following (underlined) clarification into the first sentence. “*Diagnostic network optimization (DNO), a geospatial optimization technique, can improve access to diagnostics and reduce costs through informing policy-makers’ decisions on diagnostic network changes*.”

*6. Table 3: the row of the proportion of EID tests performed on-site. The difference between 52% and 11% is 41%. Not 36% as it is now.*

**Response:** Thank you for picking this up. This has been corrected in the manuscript.
